# *Semen Trigonellae* alleviates LPS‐induced depressive behavior via enhancing the abundance of *Ligilactobacillus* spp.

**DOI:** 10.1002/fsn3.4475

**Published:** 2024-10-10

**Authors:** Wenhui Chang, Jing Guo, Yanan Yang, Linen Zou, Yu Fu, Mingxi Li, Leilei Li, Chenxi Li, Xinya Wang, Xiaohui Zhao, Chongming Wu

**Affiliations:** ^1^ School of Chinese Materia Medica Tianjin University of Traditional Chinese Medicine Tianjin China; ^2^ Tianjin Key Laboratory of Therapeutic Substance of Traditional Chinese Medicine Tianjin China

**Keywords:** depression, gut microbiota, *Ligilactobacillus animalis*, *Ligilactobacillus murinus*, *Semen Trigonellae*

## Abstract

The rising incidence rate of depression presents a substantial threat to human well‐being. *Semen Trigonellae* (ST), the dried mature seeds of *Trigonella foenum‐graecum* L., has a long‐standing traditional reputation for alleviating anxiety and hopelessness. However, the anti‐depressant mechanism of ST remains poorly understood. This study aimed to assess ST's anti‐depressant, as well as explore its potential mechanism from a gut microbial aspect. The Kunming mice were challenged by lipopolysaccharide (LPS) to induce depression‐like behavior and then orally administrated with aqueous extract of ST. The behavioral test, and hippocampal and serum biochemical indicators were detected to assess anti‐depressant effect of ST. We utilized full‐length 16S rRNA gene sequencing to investigate how ST influences gut microbiota modulation. Administration of ST mitigated LPS‐induced depression‐like behaviors. ST reversed the decrease in hippocampal 5‐hydroxytryptamine (5‐HT) and brain‐derived neurotrophic factor (BDNF) levels while reducing serum levels of tumor necrosis factor α (TNF‐α), interleukin 1β (IL‐1β), and interleukin 6 (IL‐6). Microbial analysis revealed that administration of ST markedly shifted the gut microbiota structure, dramatically and dose‐dependently increased the abundance of *Ligilactobacillus murinus* and *Ligilactobacillus animalis*. Experimentally, oral administration of live *L*. *murinus* and *L*. *animalis* to LPS‐challenged mice yielded similar effects to ST in ameliorating depression, elevating 5‐HT and BDNF, and reducing proinflammatory cytokines. These findings provide evidence that ST is a promising medical food for the management of depression, in which modulation of the gut microbiota, particularly enhancing the abundance of *Ligilactobacillus* plays an important role.

## Graphical abstract

*Semen Trigonellae* (ST) markedly attenuates the lipopolysaccharide (LPS)‐induced depressive‐like behaviors. *Semen Trigonellae* notably enriches *Ligilactobacillus* spp. in LPS‐induced mice. *Ligilactobacillus murinus* and *L*. *animalis* attenuate the LPS‐induced depressive‐like behaviors. Gut microbiota mediates the anti‐depressive effect of ST.

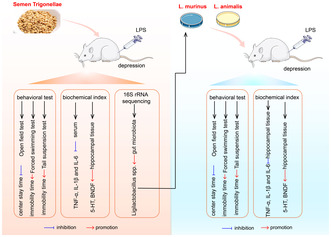

## 1 INTRODUCTION

Depression, a widespread psychiatric condition, significantly affects a considerable number of individuals across the globe. Its ramifications extend far beyond mental well‐being to physical health and healthcare systems. Epidemiological research indicates that depression's prevalence within the general population varies between 10% and 15% (Patil et al., 2023). In China, the estimated lifetime occurrence of depression among adults stands at 6.9% (Li et al., 2022), with roughly two‐thirds of those affected showing signs of suicidal tendencies. Drug therapy serves as the primary treatment approach for depression, with psychological counseling often used as a complementary form of treatment (Patil et al., 2023). However, current pharmaceutical interventions for depression demonstrate effectiveness in only about half of instances, while an additional one‐third of patients fail to exhibit improvement with medication (Zhang et al., 2017). Consequently, the need to discover more effective interventions for depressive disorders becomes increasingly urgent.

Traditional Chinese medicines (TCMs), particularly food‐medicine dual TCMs, offer distinct advantages in terms of safety, efficacy, and comprehensive regulation (Xu et al., 2024; Yang & Wu, 2022; Zhang et al., 2020; Zhong et al., 2024). *Semen Trigonellae*, the dry mature seed of the *Trigonella foenum‐graecum* L. (Leguminosae), possesses rich phytochemical concentration (Agrawal et al., 2023). Historically, this specific seed has fulfilled diverse functions within the realm of medicinal nutrition, encompassing areas such as dietary sustenance, nutraceutical applications, medicinal purposes, and therapeutic interventions. Its extensive use in traditional medicine dates back to ancient times, and its components have traditionally been used in the management of liver injury, cancer, diabetes, and neurological disorders (Chen et al., 2022; Gavahian et al., 2023). Saponin isolated from *Semen Trigonellae* has demonstrated the ability to inhibit apoptosis, suppress acetylcholinesterase (AChE) activity, and regulate malondialdehyde (MDA) levels, thereby potentially influencing the development of Parkinson's disease (Visuvanathan et al., 2022). Moreover, the administration of *Semen Trigonellae* extract to patients with Alzheimer's disease (AD) has demonstrated promising outcomes in terms of memory enhancement, dissipation of depressive emotions, overall quality of life improvement, blood pressure regulation, and reduction of oxidative stress. (Foroumandi et al., 2023). Notably, a randomized, double‐blinded, placebo‐controlled clinical trial, has yielded compelling evidence supporting the effectiveness of *Semen Trigonellae* extract in alleviating depression. Concurrently, it restores hormonal equilibrium in perimenopausal women without adverse effects (Khanna et al., 2020). Regarding the anti‐depressant mechanism of *Semen Trigonellae*, Wang et al. discovered that flavonoids derived from *Semen Trigonellae* downregulated the Kruppel‐like factor11 (KLF11)/Silent mating type information regulation 2 homolog 1 (SIRT1)‐monoamine oxidase A (MAO‐A) signaling pathway, this led to the suppression of MAO‐A expression and enhancement of monoamine neurotransmitter activities, ultimately eliciting antidepressant effects in mice under chronic restraint stress (Wang et al., 2019). However, the available data on other mechanisms of *Semen Trigonellae*'s anti‐depressant properties remain limited due to the multi‐targeted nature of TCMs.

While the precise mechanisms driving the onset and progression of depression are still not fully understood, recent findings point to a notable involvement of the gut microbiota (Duan et al., 2022). Serving as the body's largest virtual organ, the gut microbiota actively engages in numerous physiological processes and facilitates bidirectional brain‐intestine communication via the gut‐brain axis (Basiji et al., 2023; Yang et al., 2023). Alterations in gut microbiota can influence intestinal barrier permeability, which serves as the primary defense mechanism that separates the gut from the external environment. Similarly, the gut microbiota composition also impacts the activity and function of the hypothalamic–pituitary–adrenal (HPA) axis (Zhao et al., 2023). This subsequently influences the regulation and release of monoamine neurotransmitters and brain‐derived neurotrophic factor (BDNF), as well as immune responses and systemic inflammation (Basiji et al., 2023; Yang et al., 2023). According to a clinical study, individuals with depression exhibit a distinct microbial community compared to those without depression. This distinction is primarily characterized by an increase in *Oscillibacter*, *Blautia*, *Holdemania*, *Clostridium XIX*, *Anaerostipes*, *Lachnospiracea incertaesedis*, *Anaerofifilum*, *Streptococcus*, *Gelria*, *Turicibacter*, *Parabacteroides*, *Eggerthella*, *Klebsiella*, *Streptococcus*, *Paraprevotella*, *Veillonella*, and *Clostridium IV* and a decrease in *Coprococcus*, *Lactobacillus*, *Clostridium XlVa*, *Dialister*, *Howardella*, *Pyramidobacter*, and *Sutterella* (Barandouzi et al., 2020). *Lactobacillus* and *Bifidobacterium*, renowned representatives of probiotics, have been substantiated to enhance the expression level of tryptophan hydroxylase 1 (TPH1) in the intestine. This augmentation leads to an increased synthesis of 5‐hydroxytryptophan (5‐HTP), subsequently promoting the release of 5‐hydroxytryptamine (5‐HT) (Tian et al., 2022; Xie et al., 2020). Furthermore, these probiotics also contribute to immune system homeostasis, by reducing CD4(+) T cells differentiation into pro‐inflammatory T cells and mitigating the inflammatory response of central microglia, thereby attaining anxiolytic and anti‐depressive outcomes (Tian et al., 2022; Xie et al., 2020). Based on these investigations, orienting therapeutic strategies toward modulation of the gut microbiota holds considerable promise as a means to bolster the effectiveness of pharmacotherapy for depression management.

Despite numerous studies conducted over the years exploring the antidepressant potential of *Semen Trigonellae*, the precise mechanisms driving its effects remain largely elusive. This is attributed to the intricate and heterogeneous of the disease etiology itself. By conducting this study, our objective is to authenticate the anti‐depressant effect of *Semen Trigonellae* in mice with LPS‐induced depression‐like behavior. Furthermore, we have explored the mechanistic aspects of how *Semen Trigonellae* modulates gut microbiota and identify the specific microbial species involved in its anti‐depressant effect. This study has yielded significant findings, enabling the identification of a novel mechanism by which *Semen Trigonellae* exerts a promising positive impact on depression through gut microbiota.

## 2 MATERIALS AND METHODS

### 2.1 Preparation of *Semen Trigonellae* aqueous extract

*Semen Trigonellae* was purchased from Hainan Zhengshengtang Health Industry Group Co., Ltd., authenticated by Professor Niankai Zeng, and deposited at Hainan Medical University. The *Semen Trigonellae* aqueous extract was prepared according to the previously established protocol (Yang et al., 2022). In brief, 100 g of dried *Semen Trigonellae* underwent crushing and extraction with 1000 mL of distilled water twice through decoction. The obtained extract was combined, filtered, and then concentrated to yield the *Semen Trigonellae* aqueous extract at a concentration of 1 g/mL (100 mL).

### 2.2 Animal experiment

#### 2.2.1 Anti‐depressant effect of *Semen Trigonellae*

All experimental procedures strictly adhered to the guidelines for the care and use of laboratory animals established by the National Institutes of Health (NIH). Approval was obtained from the Medical Ethics Committee of Tianjin University of Traditional Chinese Medicine (No. TCM‐LAEC2023083).

Fifty male SPF Kunming mice, 8 weeks, 39–43 g, were procured from Beijing HFK Bioscience Co., Ltd. The animals were accommodated in air‐conditioned room at the Animal Center (20–25°C, 40–50%, 12:12 h dark/light), and fed with a regular chow diet and tap water. After 1 week of acclimatization, mice were randomly assigned to five groups: the control group (Control), the LPS group (LPS), the fluoxetine group (fluoxetine), the low‐dose *Semen Trigonellae* group (ST‐L), and the high‐dose *Semen Trigonellae* group (ST‐H). During the first two‐week treatment period, the mice in control and LPS groups were orally administered with equal amounts of distilled water, the mice in fluoxetine group were orally administered with fluoxetine aqueous solution (3 mg/kg), while mice in ST‐L and ST‐H group were orally administered with low‐dose (400 mg/kg) and high‐dose (800 mg/kg) of *Semen Trigonellae* aqueous extract, respectively. Following a two‐week treatment, all groups except the control group underwent an intraperitoneal injection of LPS (1 mg/kg) to induce a model of depression. After 24 h, the open field test and tail suspension test were carried out according to the established protocol. Two days later, another intraperitoneal injection of LPS was administrated, and the forced swimming test was undertaken 24 h later. During LPS injection and behavioral experiments, ST and fluoxetine were continuously given to mice. On the 21st day, each mouse's body weight was recorded and subsequently executed. Blood samples were taken to extract serum. Furthermore, hippocampal tissue and freshly collected fecal samples were stored at −80°C for subsequent analysis.

#### 2.2.2 Anti‐depressant effect of *Ligilactobacillus murinus* and *Ligilactobacillus animalis*

**FIGURE 1:**
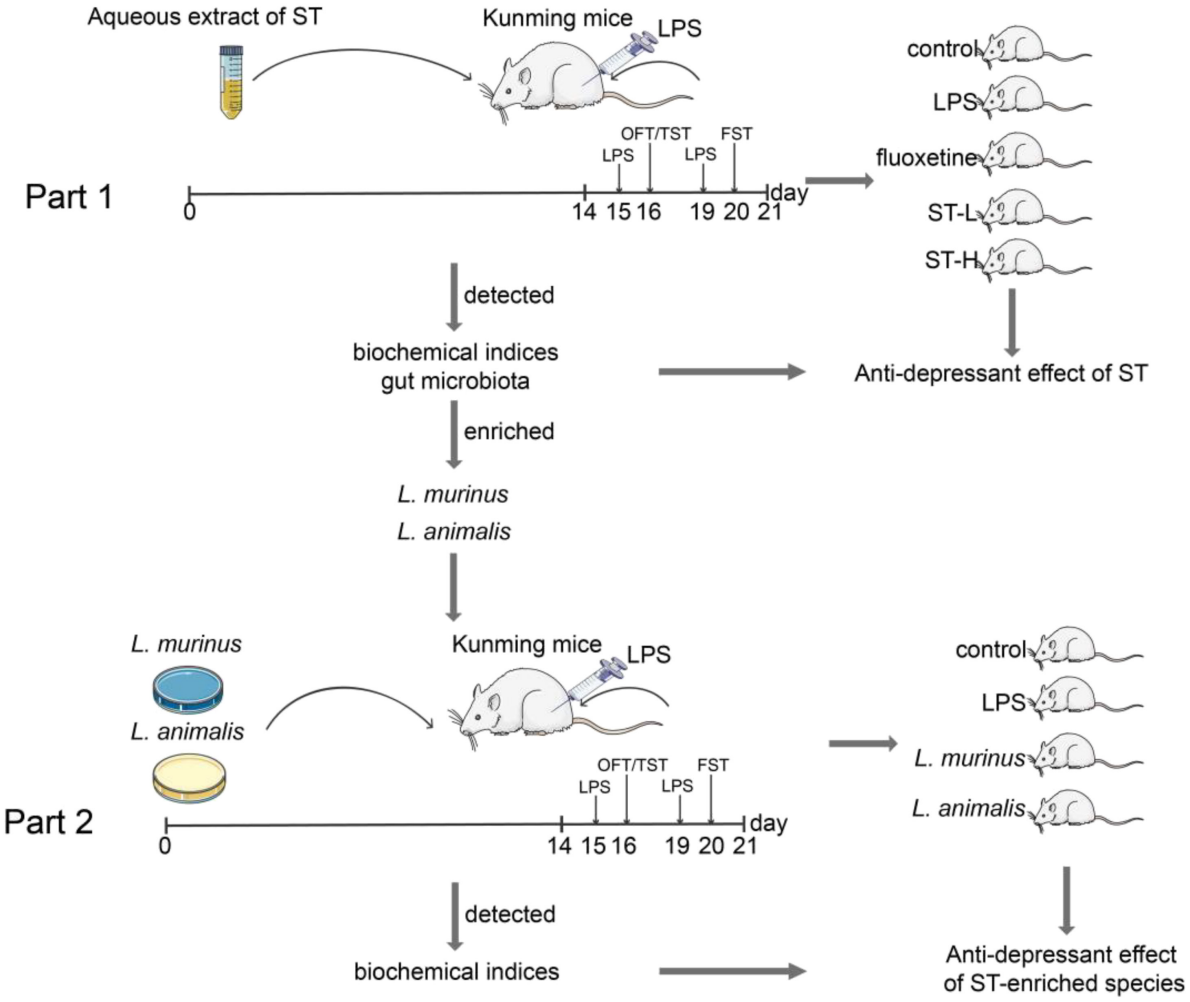
The study design of animal experiment.

To further confirm the anti‐depressant function of ST‐enriched *L*. *murinus* and *L*. *animalis*, we conducted an additional animal experiment (Figure 1). In this experiment, 40 male SPF Kunming mice, 39–43 g, were provided by Beijing HFK Bioscience Co., Ltd. After 1 week of acclimatization, mice were randomized equally into four groups: the control group (Control), the LPS group (LPS), the *Ligilactobacillus murinus* group (*L*. *murinus*), and the *Ligilactobacillus animalis* group (*L*. *animalis*). During the first two‐week treatment period, *L*. *murinus* and *L*. *animalis* groups were intragastric administrated with suspensions containing corresponding probiotics (10^9^ CFU/day/mice), while the control and LPS groups were orally administered with equal amounts of distilled water. After 2 weeks of treatment, the depression model and behavioral tests were performed as mentioned above. After 3 weeks, the mice were euthanized, and serum and hippocampal tissues were collected.

### 2.3 Open field test

As mentioned earlier, the open field test (OFT) was administered 24 h after the last LPS injection (Xu et al., 2023). These mice were moved to the behavioral laboratory and kept for 1 h to acclimate to their surroundings. Subsequently, they were placed in a plastic box for 5 min, during which their spontaneous movement trajectories, including the center dwell time of each mouse were recorded. This experiment should be conducted in a dark environment and the box should be cleaned with 75% ethanol to avoid the influence of light and odor.

### 2.4 Forced swimming test

Similarly, the forced swimming test (FST) was administered as instructed (Xu et al., 2023). Firstly, mice were transferred to the behavioral laboratory and allowed to acclimate for 1 h. The mouse was introduced into a swimming tank to swim (water temperature: 23–25°C, water depth: 30 cm) for 6 min. During the last 4 min of the test, the immobility duration was documented. The state of immobility in mice was observed when they ceased their struggle and maintained a motionless floating position in the water, only exhibiting the essential movements to keep their head above the water's surface.

### 2.5 Tail suspension test

The tail suspension test (TST) was administered as previously described methods (Xu et al., 2023). Briefly, after acclimation to the environment, the distal portion of the mice's tails was suspended using a 10 cm piece of tape affixed to a rubber tube at the top of the apparatus. The immobility time was recorded during the last 4 min of a 6 min suspension period.

### 2.6 Enzyme‐linked immunosorbent assay

Serum samples underwent enzyme‐linked immunosorbent assay (ELISA) analysis following kit instructions (Solaibao) to quantify TNF‐α, IL‐1β, and IL‐6. Additionally, hippocampal tissue was homogenized, and the resulting supernatant was utilized for ELISA to evaluate TNF‐α, IL‐1β, IL‐6, 5‐HT, and BDNF expression.

### 2.7 16S rRNA amplification sequencing

The fecal samples were subjected to extract the total bacterial DNA using XFastDNA® Spin Kit for Stool (MP Biomedicals) (Li et al., 2018). The V1–V9 region of the bacteria 16S ribosomal RNA gene was amplified by PCR (95°C for 2 min, followed by 27 cycles at 95°C for 30 s, 55°C for 30 s, and 72°C for 60 s and a final extension at 72°C for 5 min) (Wu et al., 2019) using primers 27F 5′‐AGRGTTYGATYMTGGCTCAG‐3′ and 1492R 5′‐RGYTACCTTGTTACGACTT‐3′, where barcode is an eight‐base sequence unique to each sample. PCR reactions were performed in triplicate 20 μL mixture containing 4 μL of 5 × FastPfu Buffer, 2 μL of 2.5 mM dNTPs, 0.8 μL of each primer (5 μM), 0.4 μL of FastPfu Polymerase, and 10 ng of template DNA. Amplicons were extracted from 2% agarose gels and purified using the AxyPrep DNA Gel Extraction Kit (Axygen Biosciences) according to the manufacturer's instructions. Raw sequencing reads underwent quality control, noise reduction, and the removal of chimera reads using Qiime 2. Subsequently, high‐quality sequences were clustered into operational taxonomic units (OTUs) based on a 97% sequence similarity threshold. The α‐diversity and β‐diversity were analyzed using vegan package of R software (Version 4.3.8). The α‐diversity was visualized based on Chao1, Shannon, and Ace indices, and principal coordinate analysis (PCoA) was visualized based on Bray‐Curtis distance. Differential bacteria were identified by Wilcoxon tests.

### 2.8 Statistical analysis

GraphPad Prism 8 was employed for statistical analysis. Data were presented as mean ± SEM, and figures were generated using the same software. One‐way ANOVA was utilized for normally distributed data, whereas the Wilcoxon test was applied for non‐normally distributed data. Statistical significance was established at *p* < .05.

## 3 RESULTS

### 3.1 *Semen Trigonellae* attenuates depressive‐like behavior in LPS‐induced mice

**FIGURE 2:**
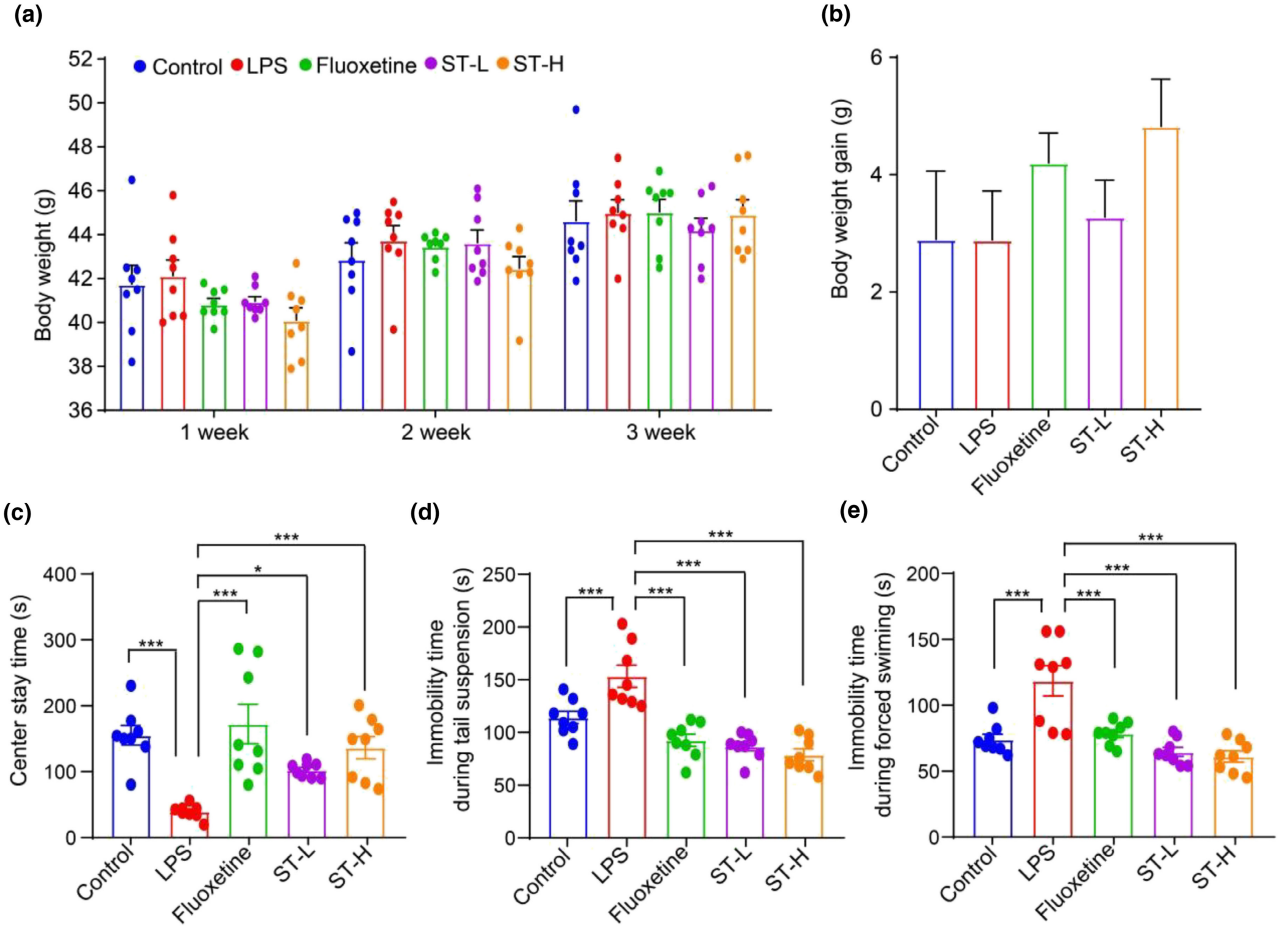
*Semen Trigonellae* (ST) attenuates depressive‐like behavior in lipopolysaccharide (LPS)‐induced mice. (a) The body weight and (b) body weight gain of mice after ST treatment. (c) Center stay time (s) of mice after LPS and ST treatment. (d) Immobility time during tail suspension (s) and (e) forced swimming of mice after LPS and ST treatment. *p < .05, ***p < .001.

Each mouse's weight was monitored weekly during the experiment. LPS injection led to an increasing trend in body weight, whereas fluoxetine or *Semen Trigonellae* treatment during the first week led to a decreasing trend in body weight compared to the control group. However, in the third week, the weight of mice in all groups was similar (Figure 2a,b). Intraperitoneally injection of LPS‐induced depression‐like behaviors, which were accompanied by a notable decrease in center stay time (Figure 2c) and an elevation in immobility duration observed during the tail suspension and forced swimming tests (Figure 2d,e). Anti‐depressant drug fluoxetine significantly reversed these changes, and administration of *Semen Trigonellae* exhibited a similar effect to fluoxetine (Figure 2c–e). These findings indicate that *Semen Trigonellae* may potentially play a role in mitigating LPS‐induced depressive symptoms in mice.

### 3.2 *Semen Trigonellae* increases hippocampal neurotransmitters and decreases serum inflammatory cytokines

**FIGURE 3:**
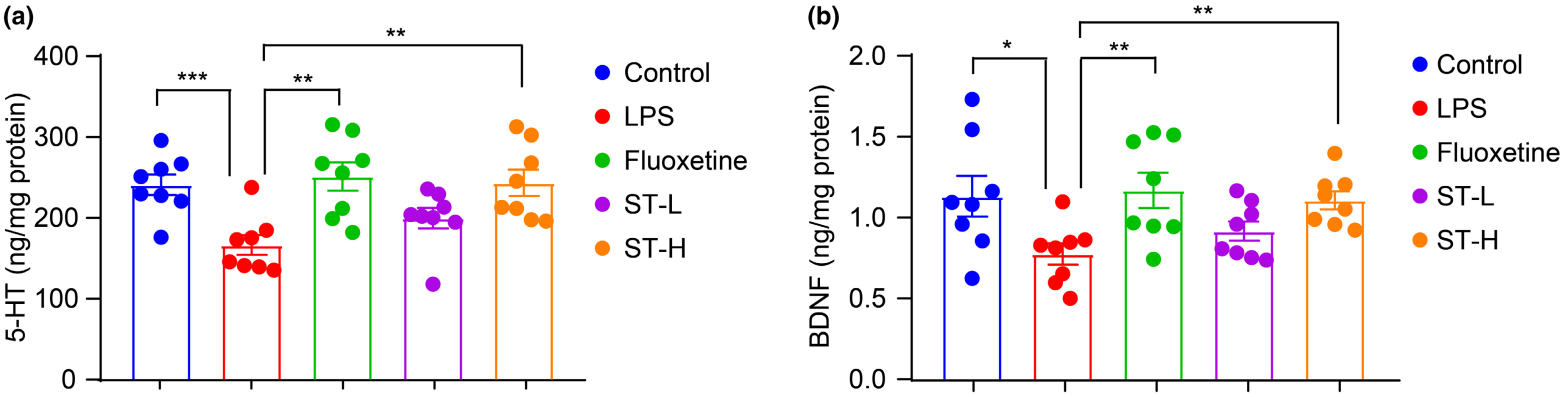
*Semen Trigonellae* restores lipopolysaccharide (LPS)‐induced decline of hippocampal neurotransmitter. The hippocampal levels of (a) 5‐HT and (b) BDNF. *p < .05, **p < .01, ***p < .001.

**FIGURE 4:**
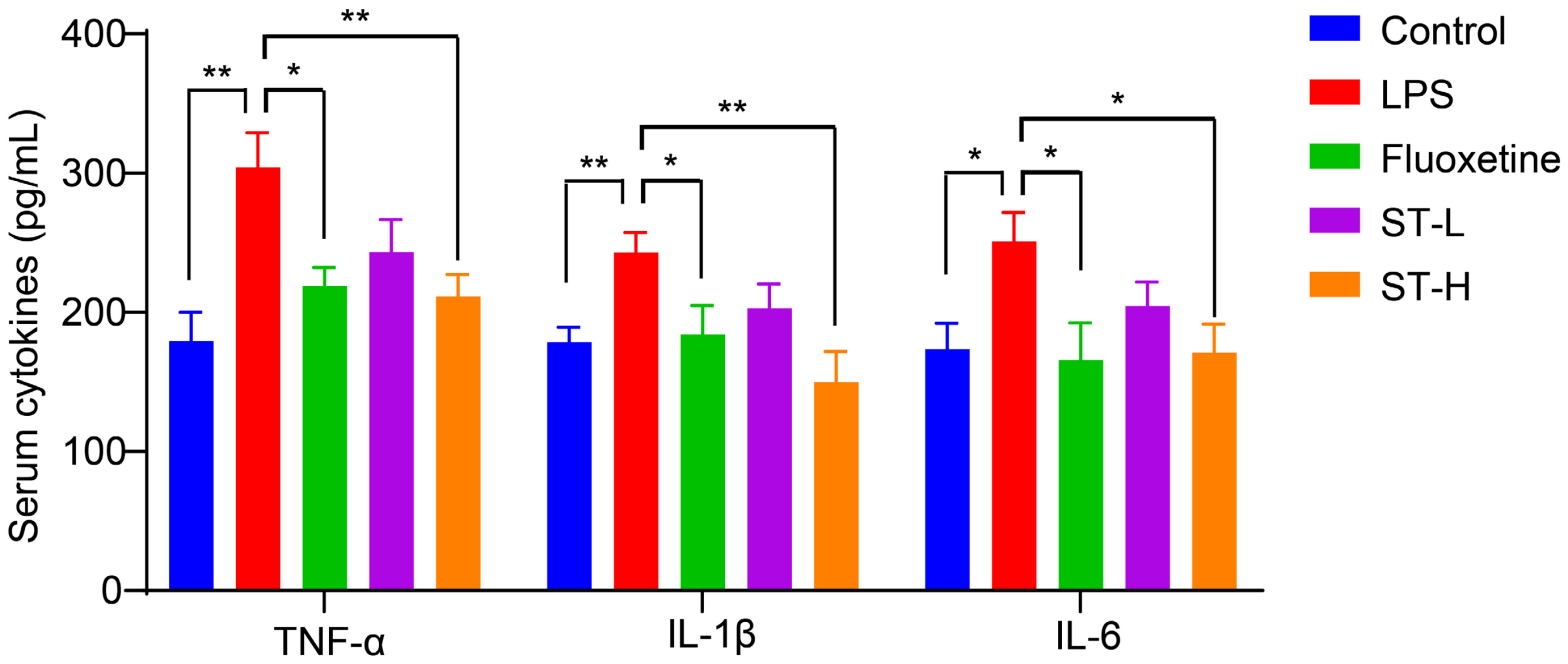
*Semen Trigonellae* prevents lipopolysaccharide (LPS)‐induced increase of hippocampal neurotransmitter. The serum levels of TNF‐α, IL‐1β, and IL‐6. *p < .05, **p < .01.

To further verify *Semen Trigonellae*'s anti‐depressant effect in LPS‐induced mice, we measured hippocampal levels of 5‐HT and BDNF. Our findings revealed a significant reduction in both 5‐HT and BDNF levels following LPS injection (Figure 3a,b). However, treatment with fluoxetine and a high dose of *Semen Trigonellae* led to a remarkable increase in these neurotransmitters (Figure 3a,b). We also examined changes in serum cytokine levels following LPS and *Semen Trigonellae* treatment. As expected, LPS administration markedly elevated serum TNF‐α, IL‐1β, and IL‐6. Interestingly, the levels of inflammatory cytokines significantly decreased following treatment with fluoxetine and a high dose of *Semen Trigonellae* (Figure 4). These results demonstrated that the intervention with *Semen Trigonellae* may upregulate 5‐HT and BDNF levels in the hippocampus while downregulating inflammatory cytokines levels in the serum to enforce anti‐depressant effect in LPS‐induced mice.

### 3.3 *Semen Trigonellae* modifies gut microbiota diversity and structure

**FIGURE 5:**
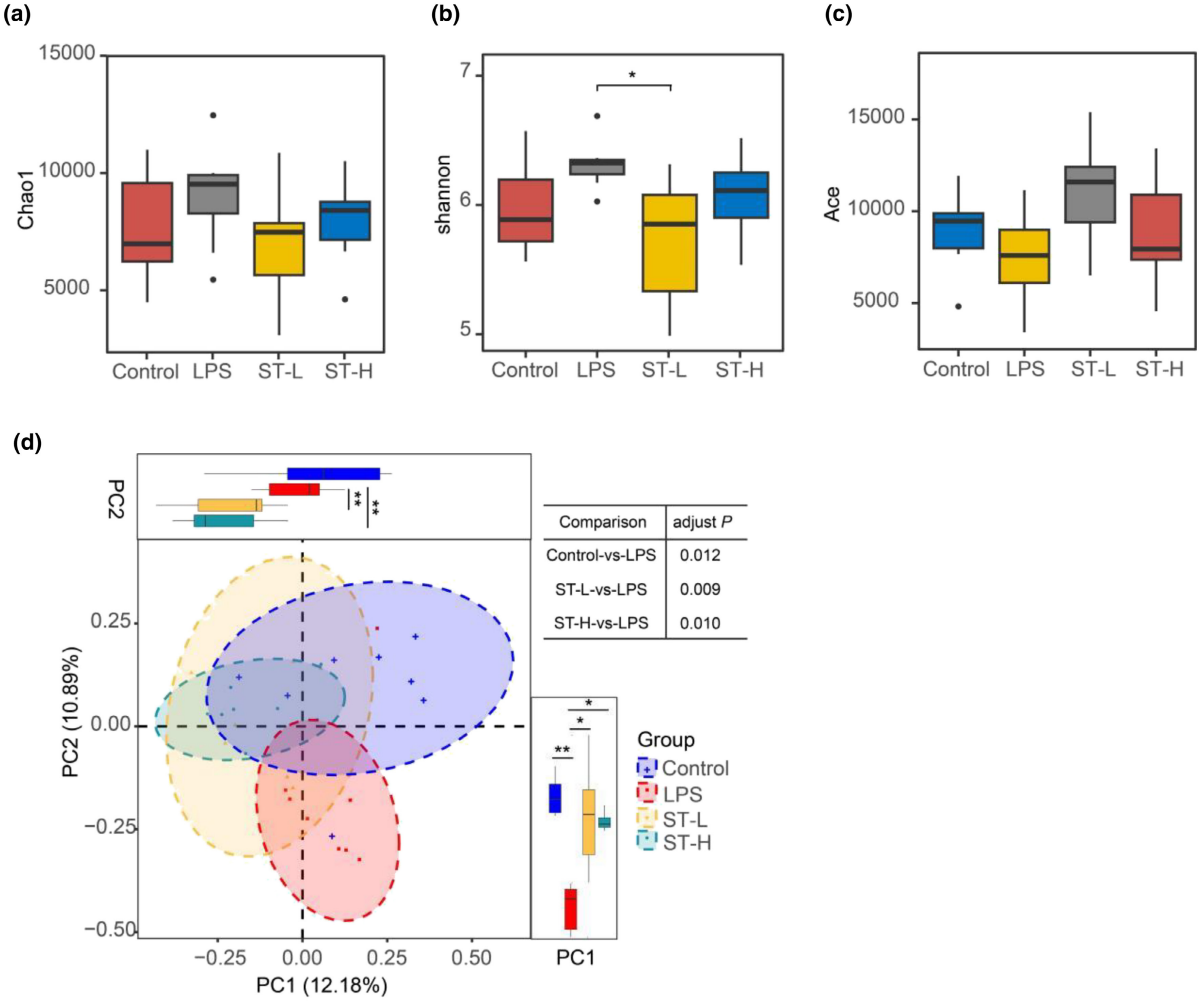
The effect of *Semen Trigonellae* on gut microbial diversity and structure. The alpha‐diversity of gut microbiota was assessed by (a) Chao1, (b) Shannon, and (c) Ace indices. (d) The structure of gut microbiota was presented by PCoA diagram. *p < .05, **p < .01.

Dysregulation of gut microbiota is considered a potential contributor to depression, influencing communication between the gut and brain via bidirectional signaling. We, therefore, dissected how *Semen Trigonellae* affects gut microbiota in LPS‐induced depressive mice. The Chao1, Shannon, and Ace indices employed to evaluate the α‐diversity of gut microbiota indicated that LPS injection led to an increasing trend of α‐diversity of gut microbiota compared to the control group (Figure 5a–c). However, the administration of a low dose of *Semen Trigonellae* led to a significant decrease in α‐diversity compared to LPS group, while a high dose of *Semen Trigonellae* only slightly reduced it without statistical differences (Figure 5a–c). Principal coordinate analysis (PCoA) revealed that LPS disrupted gut microbiota structure, whereas *Semen Trigonellae* shifted it closer to a normal state. PCo1 accounted for 12.18% of the variation, with the *Semen Trigonellae* group closely resembling the control group, while PCO_2_ explained 10.89% of the variation, with the *Semen Trigonellae* group diverging from the control group (Figure 5d).

### 3.4 *Semen Trigonellae* enriches *Ligilactobacillus* spp. in LPS‐induced mice

**FIGURE 6:**
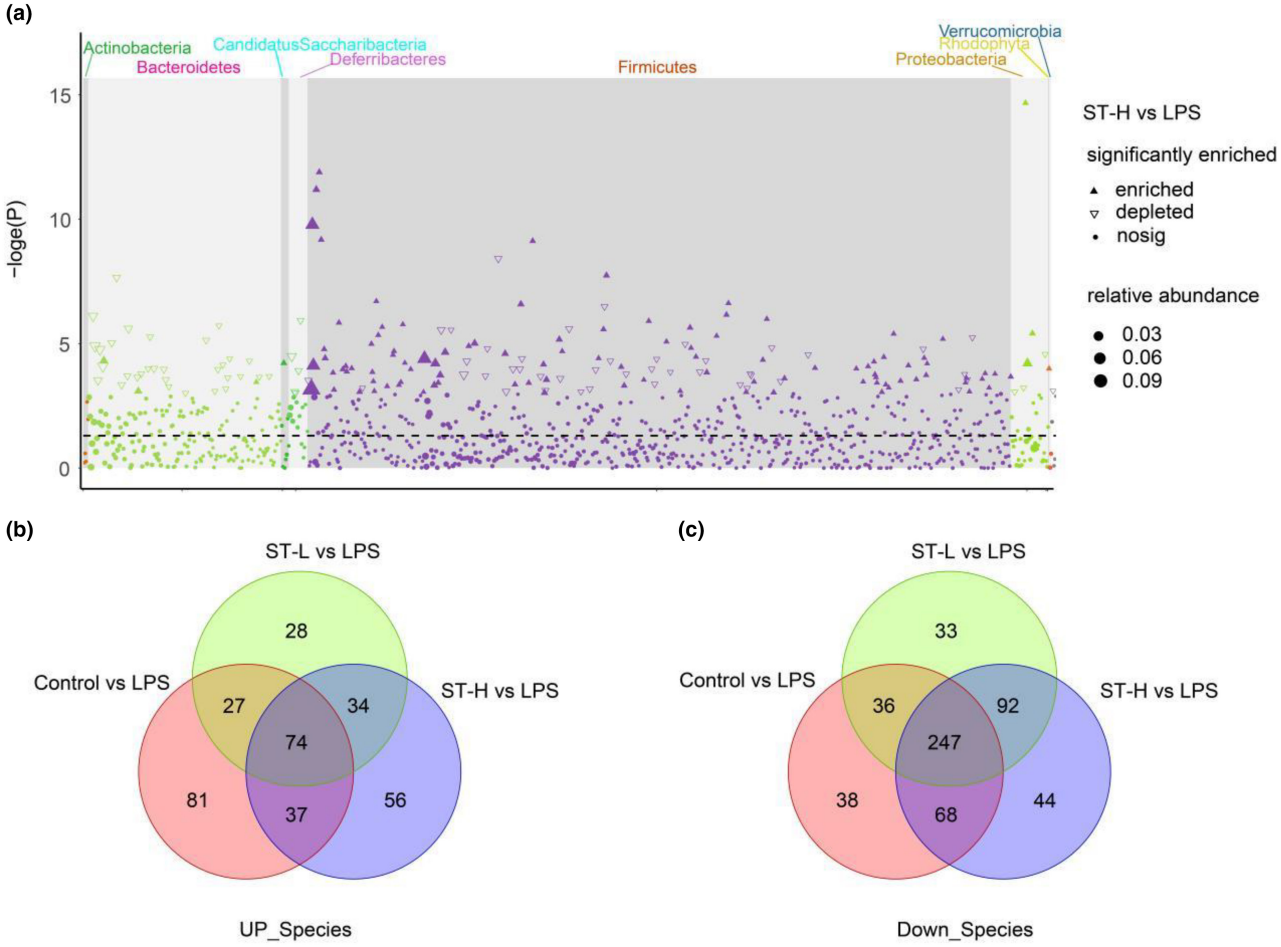
The composition change of gut microbiota after *Semen Trigonellae treatment*. (a) Manhattan plot of gut microbial phylum. (b) Venn diagrams showing the upregulated and (c) downregulated species of gut microbiota within group comparison.

We next refined the analysis of gut microbiota composition to identify which genus significantly contributed to the anti‐depressant effect of *Semen Trigonellae*. Manhattan plot showed a marker enrichment of Firmicutes in both the control and *Semen Trigonellae* groups (Figure 6a and Figure S1). At the species level, low‐dose of *Semen Trigonellae* upregulated 163 species (Figure 6b) and downregulated 264 species (Figure 6c). High‐dose of *Semen Trigonellae* upregulated 219 species (Figure 6b) and downregulated 222 species (Figure 6c). In total, 74 species were enriched in the control and *Semen Trigonellae* groups compared to the LPS‐induced model group (Table S1), while 102 species were depleted in the control and *Semen Trigonellae* groups (Table S2).

**FIGURE 7:**
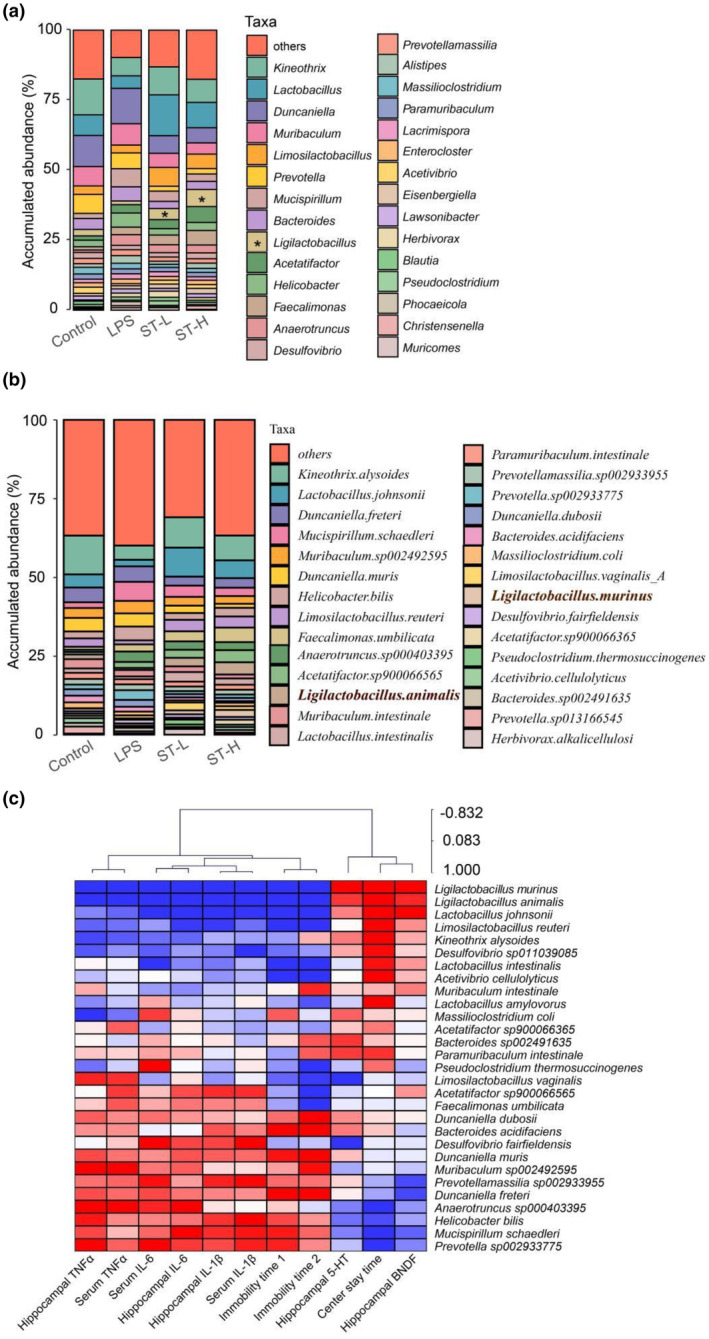
The composition change of gut microbiota. (a) The profile of genus of gut microbiota. (b) The profile of species of gut microbiota. (c) The Spearman correlation between gut microbial species and biochemical factors.

Further analysis revealed that *Semen Trigonellae* notably increased the relative frequency of the genera *Ligilactobacillus* and *Lactobacillus* (Figure 7a). Consistently, species within these two genera, including *Ligilactobacillus animalis*, *Ligilactobacillus murinus*, *Limosilactobacillus reuteri*, and *Lactobacillus johnsonii*, were markedly elevated after *Semen Trigonellae* treatment (Figure 7b). To link the relationship between gut microbiota and biochemical indices, we conducted Spearman correlation analysis on the aforementioned indices and bacteria exhibiting significant differences within each group. The heatmap revealed that *Ligilactobacillus animalis*, *Ligilactobacillus murinus*, and *Lactobacillus johnsonii* displayed positive associations with center stay time, hippocampal 5‐HT, and BDNF. Conversely, these bacteria exhibited negative relationships with serum and hippocampal TNF‐α, IL‐1β, and IL‐6, and immobility time during tail suspension and forced swimming. Similarly, *Limosilactobacillus reuteri* was positively correlated with center stay time and negatively associated with hippocampal IL‐1β and IL‐6, and immobility time during tail suspension and forced swimming (Figure 7c).

**FIGURE 8:**
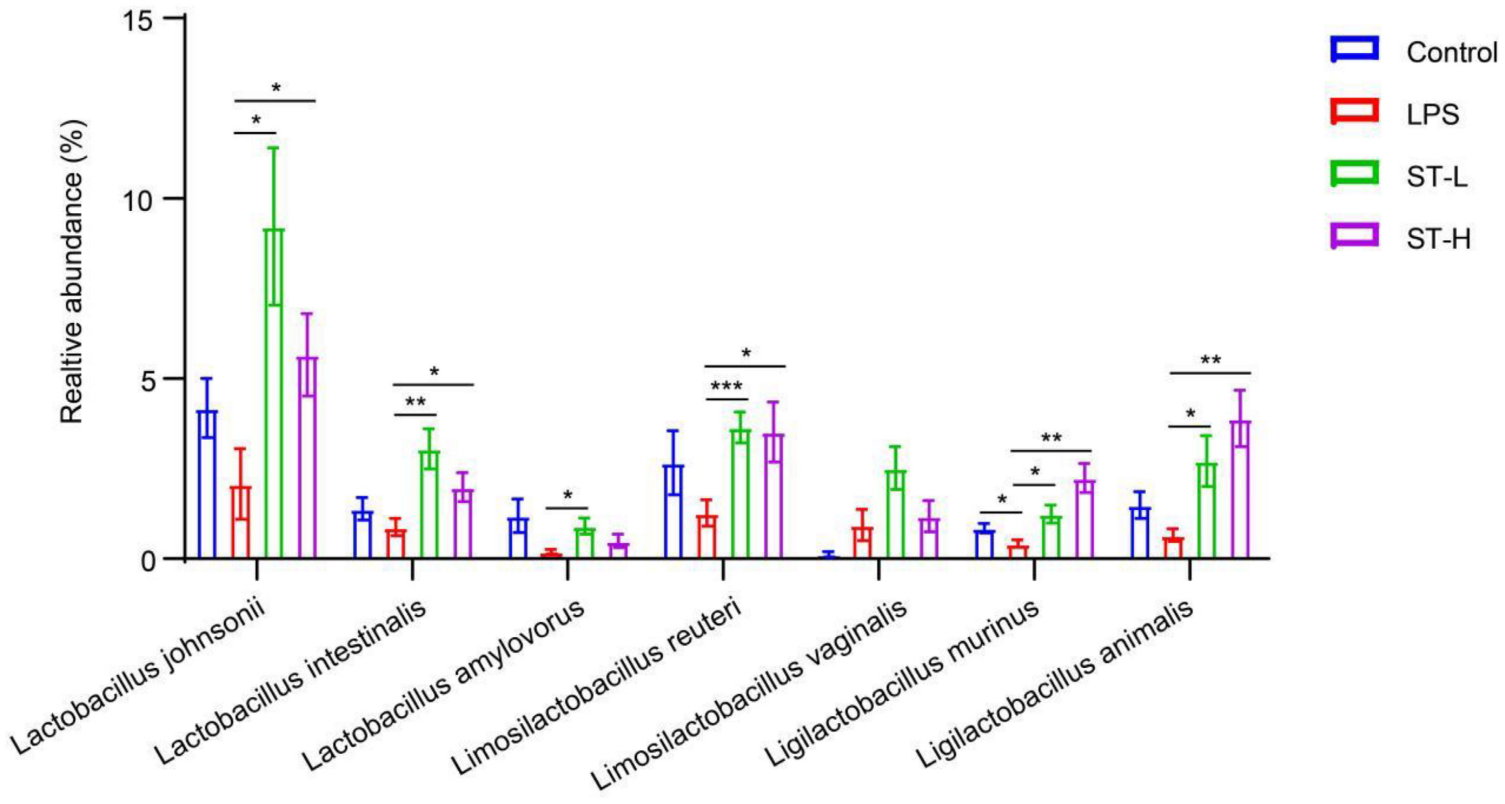
The relative abundance of *Lactobacillus*, *Ligilactobacillus*, and *Limosilactobacillus*. *p < .05, **p < .01, ***p < .001.

After treatment with *Semen Trigonellae*, we assessed the abundance of various *Lactobacillus*, *Ligilactobacillus*, and *Limosilactobacillus* species using specific primer sets. The findings indicated that the relative frequencies of *Lactobacillus johnsonii*, *Lactobacillus intestinalis*, *Lactobacillus amylovorus*, *Limosilactobacillus reuteri*, *Limosilactobacillus vaginalis*, *Ligilactobacillus murinus*, and *Ligilactobacillus animalis* significantly increased following low or high dose of *Semen Trigonellae* treatment. Notably, only *Ligilactobacillus murinus* and *Ligilactobacillus animalis* exhibited a consistent and dose‐dependent increase in abundance (Figure 8).

### 3.5 *Semen Trigonellae*‐enriched *Ligilactobacillus* alleviates depressive‐like behavior in LPS‐induced mice

**FIGURE 9:**
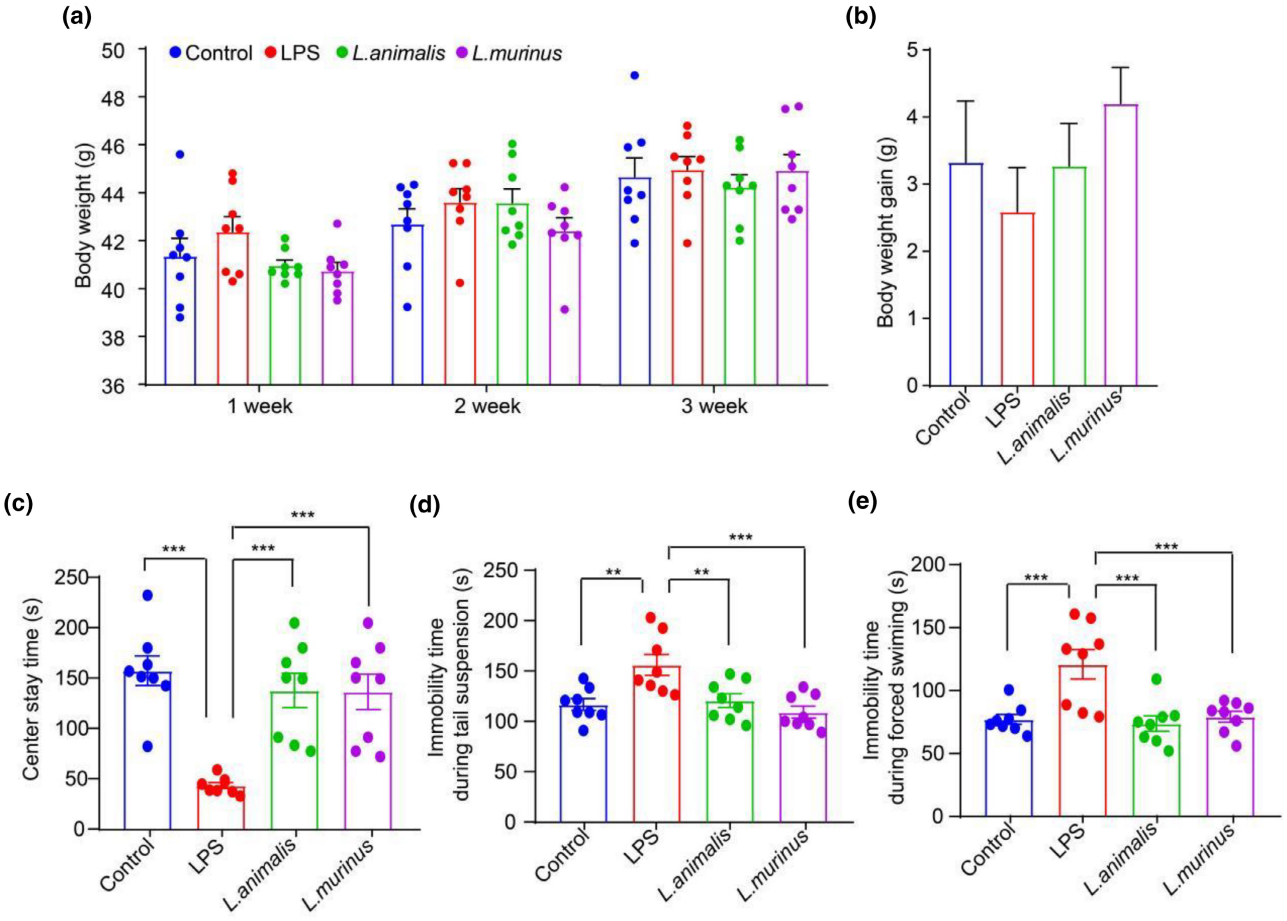
*Ligilactobacillus* alleviates depressive‐like behavior in lipopolysaccharide (LPS)‐induced mice. (a)The body weight and (b) body weight gain of mice after *L*. *murinus* and *L*. *animalis* treatment. (c) Center stay time (s) of mice after *L*. *murinus* and *L*. *animalis* treatment. (d) Immobility time (s) during tail suspension and (e) forced swimming of mice after *L*. *murinus* and *L*. *animalis* treatment. **p < .01, ***p < .001.

Consistent with the findings in mice treated with *Semen Trigonellae*, the body weight of mice induced with LPS was observed to initially increase but decreased during the first week following treatment with *Ligilactobacillus murinus* and *Ligilactobacillus animalis*. However, by the third week, there was only a small difference observed among the four treatment groups (Figure 9a). Furthermore, both *Ligilactobacillus murinus* and *Ligilactobacillus animalis* were found to notably prolong the center stay time (Figure 9b) and reduced immobility time during tail suspension and forced swimming in LPS‐induced depression mice (Figure 9c,d). These results proved that *Semen Trigonellae*‐enriched *Ligilactobacillus* could ameliorate depressive‐like behavior in LPS‐induced depressive mice.

### 3.6 *Semen Trigonellae*‐enriched *Ligilactobacillus* increases hippocampal neurotransmitters and decreases inflammatory cytokines

**FIGURE 10:**
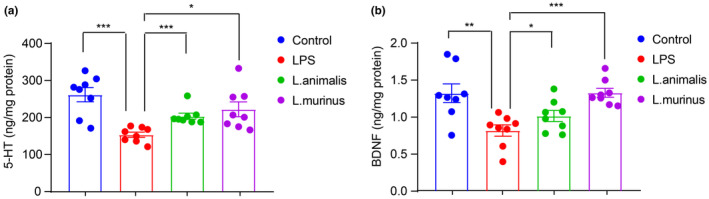
*Ligilactobacillus* prevents lipopolysaccharide (LPS)‐induced decline of hippocampal neurotransmitter. The hippocampal levels of (a) 5‐HT and (b) BDNF. *p < .05, **p < .01, ***p < .001.

**FIGURE 11:**
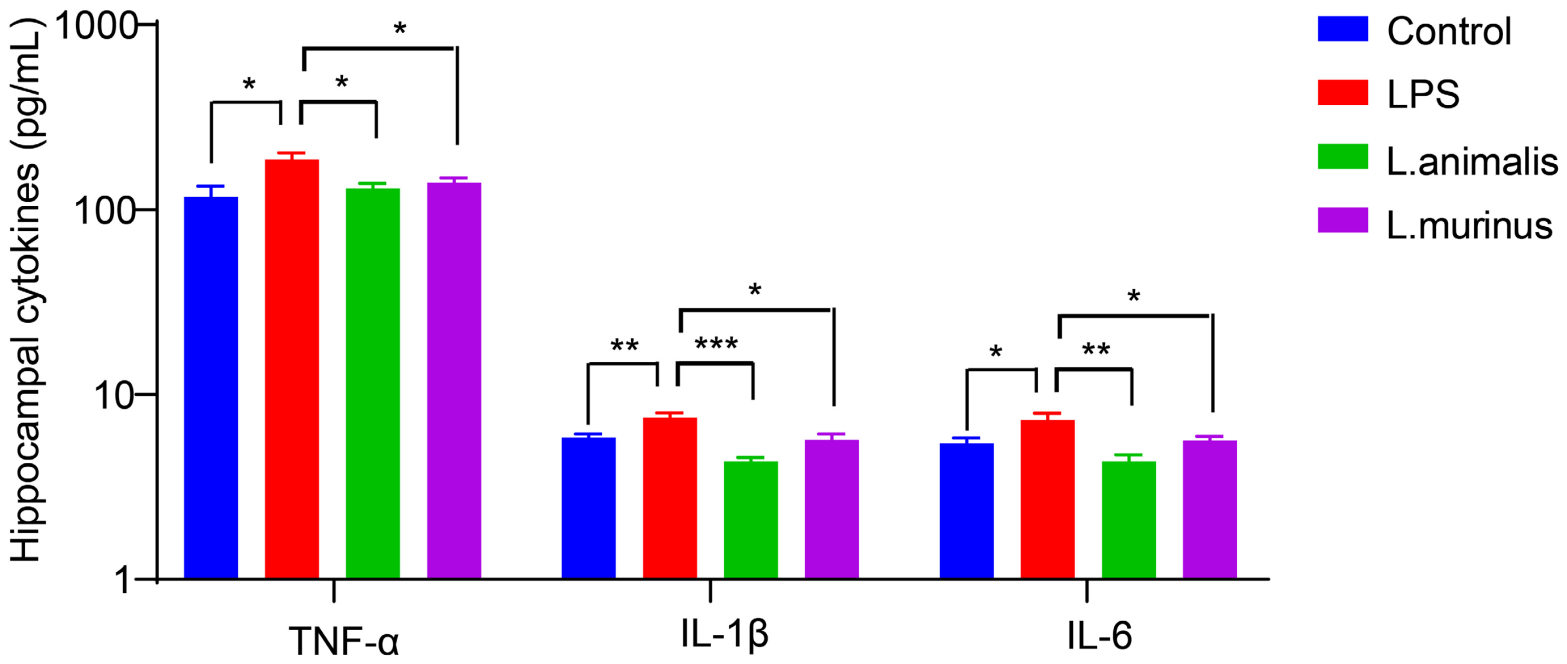
*Ligilactobacillus* prevents lipopolysaccharide (LPS)‐induced increase of inflammation. The hippocampal levels of TNF‐α, IL‐1β, and IL‐6. *p < .05, **p < .01, ***p < .001.

Next, we assessed the alteration in hippocampal neurotransmitter levels following different treatments. Remarkably, the administration of *Ligilactobacillus murinus* and *Ligilactobacillus animalis* in LPS‐induced depressant mice resulted in a considerable elevation in hippocampal 5‐HT and B levels (Figure 10a,b). Furthermore, the evaluation of hippocampal cytokines demonstrated a considerable elevation in TNF‐α, IL‐1β, and IL‐6 levels elicited by LPS, which were subsequently reversed by treatment with *Ligilactobacillus murinus* and *Ligilactobacillus animalis* (Figure 11). These findings indicate that *Ligilactobacillus* spp. may mediate the anti‐depressant effect of *Semen Trigonellae* by modulating hippocampal neurotransmitters and inflammatory cytokines.

## 4 DISCUSSION

The prevalence of psychiatric morbidity is often attributed to depression disorders, including major depressive episodes or recurrent depression, which account for 42% of cases. In accordance with evidence‐based guidelines, antidepressants currently constitute the predominant pharmacological intervention for depression treatment. Nevertheless, a significant proportion of individuals with depression either goes untreated or receives inadequate treatment, often due to limited awareness or the feeling of embarrassment, even in the aftermath of suicide attempts. In such instances, food‐medicine dual TCMs may provide an alternative approach for individuals struggling with depression (Wang et al., 2018). We, therefore, elucidated the antidepressant attributes of *Semen Trigonellae* using an LPS‐induced animal model, and examined its modulatory effect on gut microbiota, thereby providing valuable insights to further understand the underlying mechanisms involved. The findings of this study verified the induction of depression‐like behaviors by LPS, which were partially alleviated by treatment with *Semen Trigonellae*. Moreover, the enrichment of *Ligilactobacillus* by *Semen Trigonellae* demonstrated a comparable therapeutic effect against depression.

According to the record of Chinese Pharmacopeia (2020, edition), *Semen Trigonellae* is traditionally used to warm the kidneys to reinforce yang and dispel cold for pain relief. Modern pharmacological studies have shown several promising effects of *Semen Trigonellae* extract and its monomer compounds, including liver protective effect (Fatima & Masood, 2021), hypotensive effect (Amini et al., 2023), anti‐cancer effect (Alghamdi et al., 2021), lipid‐lowering effect (Heshmat‐Ghahdarijani et al., 2020), anti‐ulcerative effect (Sheethal et al., 2020), and hypoglycemic property (Kiss et al., 2019). As for the neuroprotective effect of *Semen Trigonellae*, current studies mainly focused on Alzheimer's disease (Foroumandi et al., 2023), amyotrophic lateral sclerosis (Shandilya et al., 2022), Parkinson's disease (Zameer et al., 2017), and depression (Kalshetti et al., 2015; Wang et al., 2019). However, it's worth noting that the existing research has mainly examined the anti‐depressant effect of specific isolated components from *Semen Trigonellae*, leaving the overall pharmacological effects of the medicine uncertain. To fill in this gap, we constructed a depressive animal model through LPS injection and subsequently administered *Semen Trigonellae* extract to assess its antidepressant‐like properties. As anticipated, *Semen Trigonellae* significantly mitigated depressive symptoms in LPS‐induced mice, as evidenced by prolonged center stay time and reduced immobility time observed in tail suspension and forced swimming tests. Our findings were consistent with the effect of its monomer components (Wang et al., 2019).

The chemical composition of TCMs forms the basis for their efficacy. *Semen Trigonellae* contains a variety of bioactive compounds, including alkaloids, flavonoids, steroidal saponins, and 4‐hydroxyisoleucine (Agrawal et al., 2023). Previous studies have reported that estrogen isolated from *Semen Trigonellae* could enhance the level of serotonin and the activity of 5‐HT2A to alleviate depression (Khanna et al., 2020). Also, an amino acid named (2S, 3R, 4S)‐4‐hydroxyisoleucine extracted from *Semen Trigonellae* presented potent and dose‐dependent protection against stress and depression‐like behaviors (Kalshetti et al., 2015). Moreover, flavonoids from *Semen Trigonellae* also possessed an anti‐depressant‐like effect in chronic restraint stress‐induced mice (Wang et al., 2019). Because the complexity of TCM components and their efficacies are usually achieved by synergistic action of several constituents, the antidepressant effect of *Semen Trigonellae* may be credited to the collective action of its several components. Additionally, *Semen Trigonellae* is also one of the adaptogens used in TCM to balance the dynamic system of the human body to strengthen body resistance to external stressors. This adaptogenic property might also contribute to its anti‐depressant effects (Panossian et al., 2021).

Depression is always accompanied by the abnormal secretion of neurotransmitters. Generally, the hippocampal levels of neurotransmitters are implicated in the onset of depression. We monitored the variation of 5‐HT and BDNF in LPS‐induced mice and found that *Semen Trigonellae* markedly promoted their secretion. These results were consistent with Wang's findings, which reported that flavonoids of *Trigonella Foenum‐Graecum* seeds could reverse the decrease of 5‐HT induced by chronic restraint stress (Wang et al., 2019). Simultaneously, we observed that *Semen Trigonellae* reversed the decline of BDNF caused by LPS injection. It has been reported that partial impairments in BDNF expression may cause physiological disturbances in central 5‐HT neurons (Lyons et al., 1999), suggesting that the restoration of BDNF by *Semen Trigonellae* not only benefited neuron health but also maintained the homeostasis of the 5‐HT signaling axis. Additionally, intensive attention has been directed toward the role of inflammation in depression's pathogenesis. Fluoxetine, a commonly used antidepressant, has been documented to lower serum levels of inflammatory markers including IL‐1β, IL‐6, and TNF‐α (Mojiri‐Forushani et al., 2023), suggesting that inhibition of inflammation could attenuate depression. As an alkaloid extracted from *Semen Trigonellae*, Trigonelline could also inhibit the inflammatory response via reducing hippocampal levels of NF‐κB, TLR4, and TNF‐α in LPS‐challenged rats to improve their learning and memory (Khalili et al., 2018). Similarly, we also found *Semen Trigonellae* extract substantially lower serum TNF‐α, IL‐1β, and IL‐6, implying that this herbal medicine partially exerts an anti‐depressant effect via suppressing inflammation and promoting the production of neurotransmitters.

Gut microbiota‐gut‐brain axis is increasingly acknowledged as a crucial element in depression's development, emphasizing the contribution of disrupted gut microbiota on neurotransmitter expression and neuroinflammation, which participates in depression's development. *Lacticaseibacillus rhamnosus* TF318 has been documented to exert a protective effect against depressive‐like symptoms through upregulating BDNF expression and regulating gut microbiota (Zhao et al., 2023). *Akkermansia muciniphila* colonization could enhance 5‐HT in the gut, further altering the gut‐to‐brain signal to alleviate depression (Guo et al., 2023). In light of these, we examined changes in gut microbiota after LPS injection and *Semen Trigonellae* treatment to explore the gut‐related mechanism. Despite no significant variation in gut microbiota α‐diversity among groups, the whole structure was altered by LPS injection and *Semen Trigonellae* treatment. Importantly, some microbial species were predominantly altered by *Semen Trigonellae*. Among elevated species, the relative abundance of *Ligilactobacillus animalis*, *Ligilactobacillus murinus*, *Limosilactobacillus reuteri*, and *Lactobacillus johnsonii* was significantly enhanced by *Semen Trigonellae*. It is well‐known that some probiotics, strains like *Bifidobacterium adolescentis* and *Lactobacillus reuteri*, hold promise in averting anxiety/depression via inhibiting infiltration of Iba1+ and LPS+/CD11b + cells (activated microglia) into the hippocampus and inducing hippocampal BDNF expression (Jang et al., 2019). Our correlation analysis also indicated a positive relationship between *Ligilactobacillus animalis*, *Ligilactobacillus murinus*, and *Lactobacillus johnsonii*, and center stay time, hippocampal 5‐HT, BDNF. However, these species were found to be negatively associated with serum and hippocampal TNF‐α, IL‐1β, IL‐6, and immobility time during tail suspension and forced swimming. These findings suggest that *Semen Trigonellae* may exert its antidepressant effects by enhancing the abundance of *Ligilactobacillus* to some extent.

To further validate our hypothesis, we gavaged mice with 10^9^ CFU/d/mice of *Ligilactobacillus animalis* and *Ligilactobacillus murinus*, respectively. Fortunately, we found both *Ligilactobacillus animalis* and *Ligilactobacillus murinus* alleviated the depressive‐like behavior, and prevented the decline of hippocampal neurotransmitter and increase of inflammation in LPS‐induced mice. Hu et al. also demonstrated that *Lactobacillus murinus* could activate TLR2 signaling to promote the secretion of IL‐10 from macrophages (Hu et al., 2022). In parallel, a unique species of *Lactobacillus murinus* was shown to downregulate the inflammatory cytokines like TNF‐α and inhibited the formation of IL‐8 in TNF‐α‐stimulated Caco‐2 cells, thus mediating the inflammation response of the host (Pan et al., 2018). These researches may disentangle the anti‐inflammatory effect of *Lactobacillus murinus* on LPS‐induced mice. Furthermore, a study by Ji et al. found that the TCM formula Jia Wei Xiao Yao San significantly enriched *Lactobacillus animalis*, which exhibited a positive correlation with behavioral symptoms and a negative correlation with the activation of astrocytes and microglia (Ji et al., 2022). This research indicated that alterations in *Lactobacillus animalis* may contribute to the onset of depression. Therefore, it is well‐supported to conclude that *Semen Trigonellae*‐enriched *Ligilactobacillus* has the potential to effectively mitigate LPS‐induced depression.

Indeed, this study has its limitations, and there are several promising directions for future research. Firstly, we did not validate the antidepressant effect of *Semen Trigonellae* and *Ligilactobacillus* in pseudo‐sterile mice to understand the specific role of gut microbiota in this mechanism. Furthermore, more in‐depth studies on the molecular mechanisms may facilitate understanding the anti‐depressant effect of *Semen Trigonellae*, which would provide the theoretical basis for the advancement of depression therapies. Previous research revealed that heat‐killed *Lactobacillus murinus* could diminish NLRP3 inflammasome activation in microglia and the secretion of pro‐inflammatory factors, thereby hindering the advancement of neuroinflammation (Chuang et al., 2016). This finding suggests that microbial metabolites from gut microbiota may contribute to *Semen Trigonellae*'s antidepressant effect. Therefore, multi‐omics integrative analysis may provide novel perspectives on the mechanisms underlying the pharmacological actions of TCMs like *Semen Trigonellae*.

## 5 CONCLUSION

*Semen Trigonellae*, as an edible and medicinal substance, shows potential as a therapeutic remedy for depression. Mechanistically, the adjustment of gut microbiota, particularly enhancing the abundance of *Ligilactobacillus*, contributes significantly to the anti‐depressant efficacy of *Semen Trigonellae*.

## AUTHOR CONTRIBUTIONS

**Wenhui Chang:** Formal analysis (equal); investigation (equal); methodology (equal); writing – original draft (equal). **Jing Guo:** Formal analysis (equal); writing – review and editing (equal). **Yanan Yang:** Visualization (equal); writing – original draft (equal); writing – review and editing (equal). **Linen Zou:** Formal analysis (equal); methodology (equal). **Yu Fu:** Methodology (equal). **Mingxi Li:** Writing – review and editing (equal). **Leilei Li:** Investigation (equal). **Chenxi Li:** Methodology (equal). **Xinya Wang:** Methodology (equal). **Xiaohui Zhao:** Methodology (equal). **Chongming Wu:** Conceptualization (lead); data curation (lead); formal analysis (equal); supervision (lead); visualization (equal); writing – original draft (lead); writing – review and editing (lead).

## CONFLICT OF INTEREST STATEMENT

The authors declare no known competing financial interests or personal relationships that might have influenced the work reported in this paper.

## Supporting information


Figure S1.

Table S1.

## Data Availability

The data that support the findings of this study are available on request from the corresponding author.
