# Supplementary material for: Semen Trigonellae alleviates LPS‐induced depressive behavior via enhancing the abundance of Ligilactobacillus spp
Source: Food Sci Nutr. 2024 Oct 10;12(11):9414–27. doi: 10.1002/fsn3.4475 (PMC11606864; doi:10.1002/fsn3.4475)
Supplement: Supplementary file 1 — Figure S1. [file FSN3-12-9414-s002.docx]

**Supplementary materials**


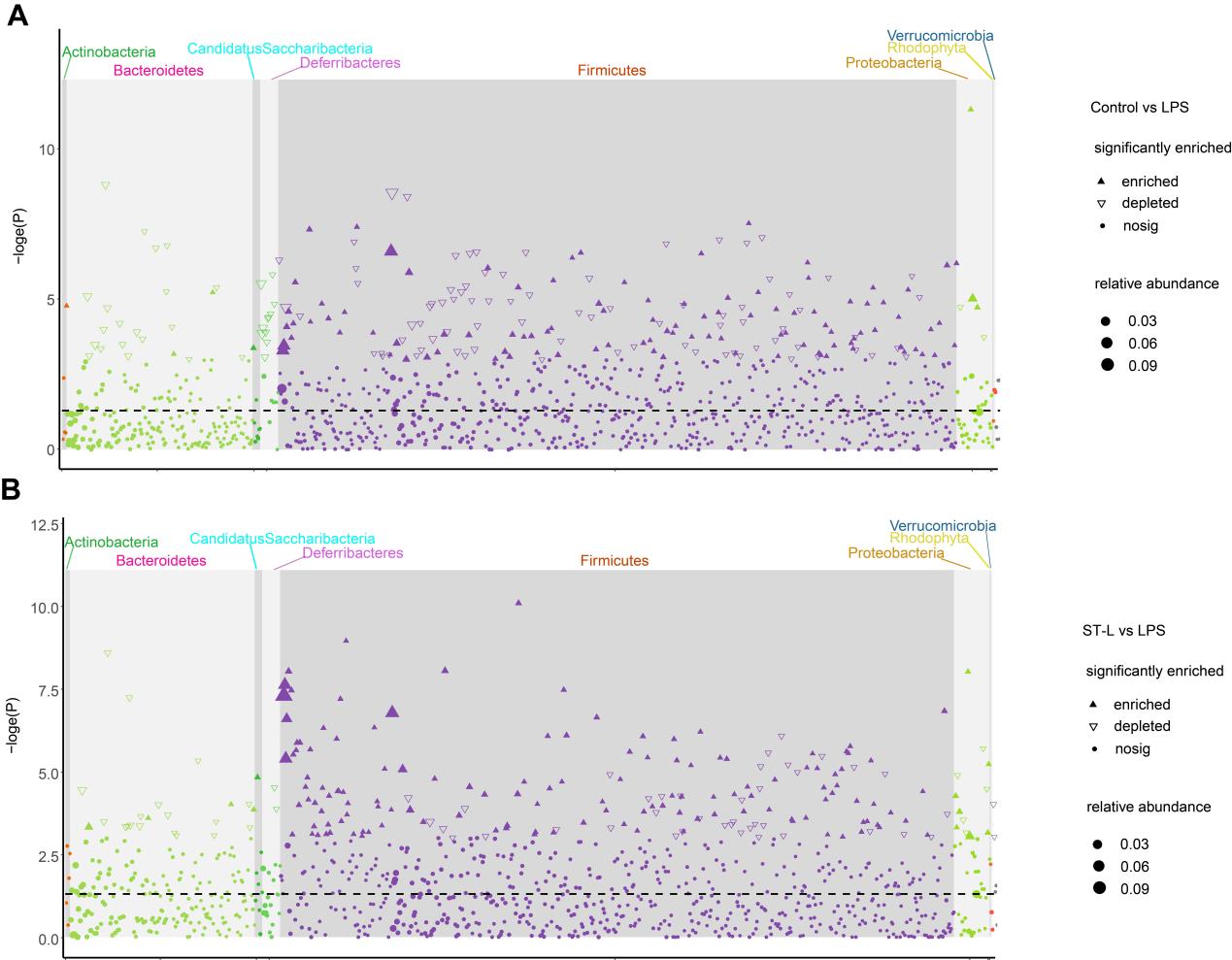


**Supplementary Figure S1.** Manhattan plot of gut microbiota at the phylum level. (A) The enriched or depleted bacteria when normal group versus LPS group. (B) The enriched or depleted bacteria when ST-L group versus LPS group.
